# Is Adapted Physical Exercise an Innovative Adjuvant Approach to Combine with Low-Protein Diet in Chronic Kidney Disease?

**DOI:** 10.3390/nu18101557

**Published:** 2026-05-14

**Authors:** Arianna Murri, Manuela Di Lauro, Elisa Grazioli, Giulia Marrone, Kevin Cornali, Luca Di Marco, Claudia Cerulli, Eliana Tranchita, Anna Paola Mitterhofer, Damiano Pietroletti, Barbara Chiaramonte, Annalisa Noce, Attilio Parisi

**Affiliations:** 1 Interdisciplinary Department of Well-Being, Health and Environmental Sustainability (BESSA), Sapienza University of Rome, via delle Fontanelle, 02100 Rieti, Italy; arianna.murri@uniroma1.it; 2 Department of Exercise, Human and Health Sciences, University of Rome “Foro Italico”, Piazza Lauro de Bosis, 15, 00135 Rome, Italy; elisa.grazioli@uniroma4.it (E.G.); claudia.cerulli@uniroma4.it (C.C.); damiano1195@gmail.com (D.P.); attilio.parisi@uniroma4.it (A.P.); 3 Department of Systems Medicine, University of Rome Tor Vergata, Via Montpellier, 1, 00133 Rome, Italy; dilauromanuela@gmail.com (M.D.L.); annapaola.mitter@uniroma2.it (A.P.M.); 4 Department of Experimental Medicine, PhD School in Biochemistry and Molecular Biology, University of Rome Tor Vergata, 00133 Rome, Italy; kevin.cornali@students.uniroma2.eu; 5 National PhD School in Kinesiology and Sport Sciences, University of Verona, Via San Francesco, 22, 37129 Verona, Italy; luca.dimarco@univr.it; 6 Sport Medicine and Hypertension Unit, Bambino Gesù Children Hospital, Piazza S. Onofrio, 4, 00165 Rome, Italy; eliana.tranchita@opbg.net; 7 UOSD Nephrology and Dialysis, Policlinico Tor Vergata, Viale Oxford, 81, 00133 Rome, Italy; 8 Department of Biomedicine and Prevention, University of Rome Tor Vergata, Via Montpellier, 1, 00133 Rome, Italy; b.chiaramonte@hotmail.com; 9 Department of Medical Sciences, Catholic University Our Lady of Good Counsel, Rr. Dritan Hoxha, 1023 Tirana, Albania

**Keywords:** chronic kidney disease, nutritional therapy, adapted physical exercise, multidisciplinary approach, quality of life

## Abstract

**Background:** In chronic kidney disease (CKD) treatment, a holistic approach, which involves not only nephrologists but also nutritionists, sports physicians, and kinesiologists, is becoming increasingly important, characterized as including not only pharmacological therapy but also integrative treatments, i.e., nutritional therapies (like low protein diet-LPD) and adapted physical exercise (APE) programs. The aim of this study was to evaluate the potential adjuvant therapeutic role of an integrated APE + LPD program on CKD comorbidities, comparing its additional beneficial effects with those induced by the LPD alone. **Methods:** This clinical study is a randomized controlled trial, where 40 CKD patients (stage G3b-G5) were enrolled and divided into two homogeneous groups: an APE + LPD group, which performed an online APE protocol combined with LPD; and an LPD group, which received only LPD. All enrolled patients were evaluated at baseline (T_0_) and after 12 weeks (T_1_) for clinical and body composition parameters and for functional assessment and health-related quality of life (HRQoL). **Results:** Both groups showed a significant reduction in lipid and glucose metabolism parameters. Good adherence to the prescribed LPD led to significantly better control of systolic blood pressure and electrolytes, along with an increase in venous bicarbonate levels. Improvements in body composition and physical performance were also observed. In the APE + LPD group only we observed a significant increase in neutrophil count, serum iron levels, muscle strength, and patients’ HRQoL. **Conclusions:** Our results suggest that the integrated approach, rather than the LPD alone, is more effective for muscle-related outcomes, HRQoL, and in the positive modulation of the immune system.

## 1. Introduction

Chronic kidney disease (CKD) is a chronic degenerative non-communicable disease whose prevalence is globally rising, and which affects around 10% of the general population [1]. By 2040, CKD is estimated to become the fifth-leading cause of death worldwide, imposing a substantial burden on national healthcare systems (NHSs) [2]. Few studies have assessed the economic burden on society of CKD patients under conservative therapy, while the analysis of direct healthcare costs, especially in end-stage kidney disease (ESKD), is more frequent. In 2021, expenditure for CKD in Italy amounted to approximately €4 billion, representing 3.2% of total healthcare expenditure borne by the NHS. Moreover, an increase in annual costs of 10.8% by 2026 has been estimated, of which renal replacement therapy, including dialysis or transplantation, accounts for 53%. Therefore, is essential to set new multidisciplinary approaches to counteract the CKD comorbidities and to not only increase lifespan but also to enhance its quality [3,4].

Several mechanisms and risk factors contribute to CKD development and progression; among these, the most frequent are cardiovascular diseases (55.9%), diabetes mellitus (42.7%), and obesity (11%). The concomitant presence of these conditions has a negative impact on quality of life (QoL) and the mortality of nephropathic patients [5]. In this context, prevention, early diagnosis, and a healthy lifestyle can significantly improve healthcare sustainability by delaying kidney damage progression and the beginning of dialysis, potentially saving hundreds of millions of euros annually and reducing the all-causes mortality risk in CKD patients [6].

In combination with the pharmacological treatment, lifestyle change is the main approach to managing CKD and slowing its progression, especially in the early stages of the disease [7,8].

Among non-pharmacological interventions, nutritional therapy and adapted physical exercise (APE) are the safest and most cost-effective ways to improve overall CKD prognosis [9,10,11,12]. When nutritional strategies are combined with APE, they can represent an adjuvant treatment for CKD itself and its comorbidities [13].

Nutritional therapy plays a key role in clinical management of CKD patients. Its main objectives are treatment of the typical signs and symptoms of CKD, delay of dialysis, and reduction of protein energy wasting (PEW) risk [12,14,15]. To achieve these goals, the nutritional therapy recommended for CKD patients, starting from stage G3b, is a low-protein diet (LPD), characterized by a protein intake of 0.6 g/kg of body weight/day, an adequate caloric intake (30–35 kcal/kg/day), a reduced intake of phosphorus and sodium, and a controlled intake of potassium [16]. Several studies suggest how the implementation of an LPD can preserve residual renal function and slow progression of the disease to end-stage kidney disease (ESKD). This is accomplished through several mechanisms, such as reducing intraglomerular pressure, lowering nitrogen (N)-derived catabolic products, improving calcium–phosphorus metabolism, counteracting endothelial dysfunction, reducing metabolic acidosis, and improving the composition of the gut microbiota [12,14,15,17]. The importance of following a proper nutritional therapy for CKD patients is so evident that several guidelines and consensus papers have been developed to guide clinicians and nutritionists towards correct nutrition.

Alongside nutritional intervention, physical exercise represents an important integrative therapeutic strategy for CKD patients, improving patients’ physical and psychological well-being [18]. The first recommendations in the field of physical activity and exercise in CKD were published in 2005 and included in the Kidney Disease Outcomes Quality Initiative (KDOQI) guidelines, concerning only hemodialysis patients [19]. In 2012, the guidelines were expanded to CKD patients under conservative therapy (stages G3–G5), suggesting at least 30 min of exercise five times a week, based on the patient’s cardiovascular status and tolerance [20]. Lastly, in 2024, the recommendations were extended to all CKD patients and suggested engaging in moderate-intensity physical activity for at least 150 min *per* week, based on the patient’s cardiovascular and physical tolerance [21]. Despite the effectiveness of lifestyle interventions, proven by scientific evidence for CKD, adherence to physical activity interventions remains low, with rates ranging from 20% to 70% [22,23]. On the other hand, sedentary behaviors are still prevalent, often due to barriers such as limited access to lifestyle programs, transportation difficulties, and other health-related issues [24]. Moreover, most of the time, kinesiologists are not included in the multidisciplinary team that manages CKD patients.

International guidelines emphasize the importance of a multidisciplinary approach to enhance patient engagement and adherence with lifestyle interventions. Physicians should collaborate with healthcare professionals to promote and support lifestyle changes during routine clinical visits, especially in the early stages of the disease [8,22,25]. A comprehensive care pathway should include not only nutritionists with expertise in CKD management but also kinesiologists to identify and implement the best practices and the most effective strategies to improve patients’ health-related quality of life (HRQoL) and, consequently, their levels of physical activity [25].

The aim of this study was to evaluate the potential adjuvant therapeutic role of an integrated APE + LPD program on CKD metabolic alterations and HRQoL, comparing its additional beneficial effects with those induced by the LPD alone. The primary endpoint was a composite assessment of sarcopenia-related outcomes, including muscle mass (quadriceps rectus femoris thickness-QRFT), muscle strength (handgrip strength test- HGST) and physical performance (short physical performance battery-SPPB) from baseline to the end of the intervention. Secondary endpoints included maintenance of body weight, amelioration of metabolic parameters, and changes in quality of life, as evaluated by the Short Form Health Survey 36 (SF-36) questionnaire. In particular, the study population was characterized by two treatment groups: one receiving only nutritional therapy and the other receiving both nutritional therapy and online supervised APE protocol, already validated [9,10,11].

## 2. Materials and Methods

This clinical study is a randomized controlled trial (RCT), where 40 CKD patients were enrolled and divided into two homogeneous groups for age, sex, and body mass index (BMI). Patient enrolment was carried out using a block randomization technique (subgroups), with each block set to a size that was a multiple of the number of treatments under study. This was done under the additional constraint of equal treatment allocation within each block. This approach ensured numerically balanced groups and maintained balance throughout the recruitment process. The randomized sequence of blocks was generated using a list of pseudo-random numbers drawn from a uniform probability distribution over a specified interval.

A blinding approach was limited on the part of the investigators. In particular, those who generated the randomization and administered the APE protocol were conscious of the treatment, while those who performed the functional assessments and clinical evaluations were blinded.

**Figure 1:**
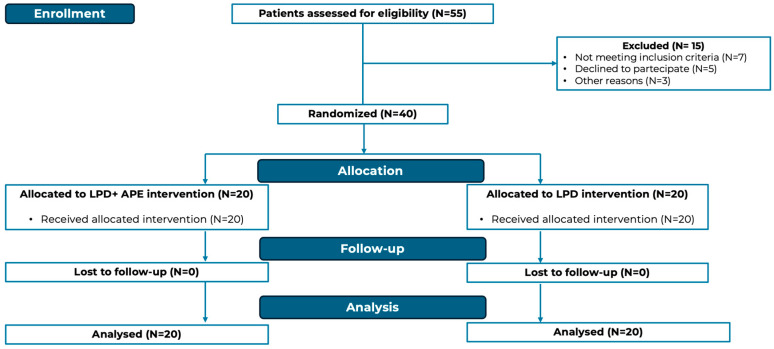
Patients’ flow diagram.

The patients’ flow diagram is shown in Figure 1.

The inclusion criteria were as follows: age between 45 and 65 years; both sexes; signature and acceptance of informed consent; CKD stage G3b-G5 (according to the 2024 Clinical Practice Guideline for the Evaluation and Management of Chronic Kidney Disease-KDIGO guidelines) [21] under conservative treatment; follow a free diet; BMI ranging from 18–35 kg/m^2^; eligible to participate in an APE program. Exclusion criteria were as follows: presence of solid or hematological malignancies; HIV, HbsAg+, HCV+ patients; non-acceptance of informed consent; patients with inflammatory and/or infectious diseases in the acute phase; presence of pregnancy and/or breastfeeding. In addition, patients were excluded if they presented a history of ischemic heart disease, including recent myocardial infarction, coronary stenting, or coronary artery bypass grafting; uncontrolled arterial hypertension; frequent or complex arrhythmia (e.g., frequent extrasystoles or atrial fibrillation); or recent cerebrovascular events (ischemic or haemorrhagic stroke).

In detail, the APE + LPD group performed 12 weeks of online APE protocol combined with a personalized LPD; and the LPD group received only a personalized LPD. The characteristics of the study population are shown in Table 1.

Patients were consecutively recruited from September 2022 to September 2024 at the Nephrology and Dialysis Unit of Policlinico Tor Vergata (PTV) in Rome.

The experimental protocol complied with the 1975 guidelines of the Declaration of Helsinki and was approved by the Independent Ethics Committee of PTV. The protocol code was 90/22 for 3 May 2022. This study was not registered on ClinicalTrials.gov, as it was conducted in Italy where national regulations do not mandate registration on that website to conduct clinical studies involving human participants.

**Figure 2:**
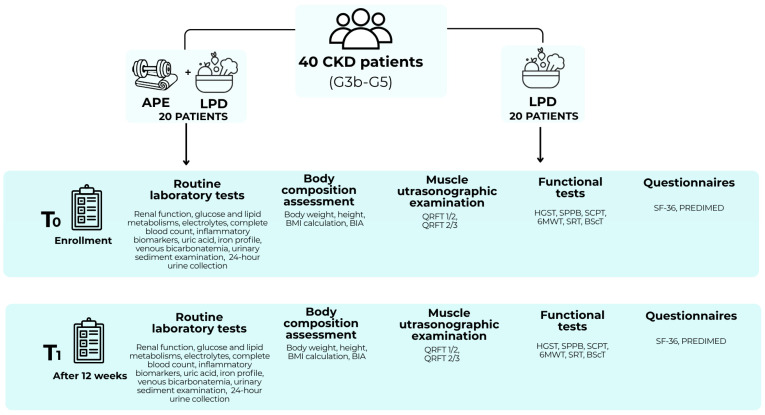
Design of the study. Abbreviations: 6MWT, six-minute walk test; APE, adapted physical exercise; BIA, bioimpedance analysis; BMI, body mass index; CKD, chronic kidney disease; HGST, handgrip strength test; LPD, low-protein diet; PREDIMED, Prevención con Dieta Mediterránea; QRFT, quadriceps rectus femoris thickness; SCPT, stair climb power test; BScT, back scratch test; SF-36, Short Form Health Survey 36; SPPB, short physical performance battery; SRT, sit and reach test.

The study’s design (Figure 2) was structured through a multidisciplinary approach involving multiple professional practitioners: the nephrologist and nutritionist for patient recruitment and dietary drawing-up; the sports physician for exercise prescription; and the kinesiologist for adapting and administering the exercise program.

*Phase 1—Patients’ enrolment and LPD prescription*

CKD patients were selected by a nephrologist, according to the above-mentioned inclusion criteria. A comprehensive medical evaluation was performed at baseline (T_0_). This included detailed medical history, routine laboratory tests, body composition evaluation, and ultrasonographic examination of QRFT. The SF-36 questionnaire was used to assess patients’ HRQoL.

Following this assessment, regardless of the sorting group, an LPD based on the consumption of protein-free products to replace conventional carbohydrate-based foods (such as bread, pasta, crispbread, biscuits) was prescribed by nephrologist, and formulated as a diet by an experienced clinical nutritionist tailored to the individual needs of each patient.

Beyond the consumption of protein-free foods, nutritionists have ensured the nutritional quality of the prescribed LPD by following the Mediterranean diet model.

Specifically, nutritional therapy was personalized and characterized by a daily caloric intake of 25–35 kcal/kg of ideal body weight and a daily protein intake of 0.6 g/kg of ideal body weight [26]. Regarding the ideal weight used to calculate caloric and protein intake, we used current weight if it corresponded to desired weight or predicted weight if this differed significantly from desired weight [27]. Sodium intake was set at 2–3 g per day, while phosphorus and potassium intakes were personalized based on the patient’s clinical characteristics [28]. Nutritional therapy was based on a weekly menu.

Based on the nutritional characteristics of the prescribed LPD, the Prevención con Dieta Mediterránea (PREDIMED) questionnaire was used to exclude potential biases arising from variations in the quality of foods included in the prescribed LPD.

The counseling activities were programmed to periodically assess adherence to nutritional therapy prescription, at an interval of every six weeks. These monitoring activities consisted of completing questionnaires aimed at assessing adherence to the prescribed diet, such as a food diary and 24-h recall. Patients’ dietary protein intake and daily sodium intake were estimated thanks to 24-h urine nitrogen and sodium excretion levels using relevant formulas reported in the scientific literature (cf. Section 2.3) [29,30,31].

*Phase 2—Sports Medicine Assessment, Kinesiologist’s Functional Evaluation, and Exercise Protocol Prescription*

At the beginning of the intervention (T_0_), the enrolled patients underwent a medical examination carried out by a sports medicine physician who performed the anamnesis to identify any risk factors and comorbidities in the CKD patients. A 12-leads electrocardiogram (ECG) at rest and systemic arterial pressure monitoring were performed to provide patients’ eligibility for physical activity and to exclude possible complications related to exercise. Exercise eligibility was determined based on the absence of uncontrolled hypertension, clinically significant arrhythmias, or ECG abnormalities suggestive of major cardiovascular disease (e.g., significant ventricular repolarization abnormalities or conduction disturbances). Patients showing unstable clinical conditions or contraindications to exercise were excluded from participation. Resting heart rate (HR_rest_) was recorded during the clinical assessment and used, together with age-predicted maximal heart rate (HR_max_ = 220 − age), to calculate heart rate reserve (HRR) according to the Karvonen formula: Target Heart Rate = [(HR_max_ − HR_rest_) × Intensity (%)] + HR_rest_.

In addition, the physician in collaboration with kinesiologists evaluated all patients through functional tests aimed at individualizing an appropriate workload and to assess baseline physical capacity and muscle strength (via SPPB and HGST, etc.). All evaluations were performed at T_0_ and T_1_. At the end of this phase, the sports physician prescribed an exercise program based on “Frequency, Intensity, Type, Timing, Volume, Progression” (FITT-VP) training parameters. This prescription defined the general training framework, which was subsequently implemented and adapted by the kinesiologist during the intervention, as follows:

- Frequency: three times a week, with a minimum of two sessions a week guaranteed.
- Intensity: aerobic training (AT) was prescribed at moderate intensity of approximately 65–70% of the HRR, while resistance training (RT) was prescribed at moderate-to-vigorous intensity, corresponding to a rating of perceived exertion (RPE) of 3–4 on the Borg CR10 scale. RPE was also used to adapt and monitor exercise intensity throughout the intervention, and in participants treated with beta-blockers, where HR was considered an unreliable indicator of exercise intensity.
- Type: a combined training program (aerobic and resistance exercises) to be performed under kinesiologist supervision and administered via online platform. For AT, body weights or any other intensity-controlled activity were performed. If AT was carried out with music, the tracks were around 120–130 bpm to guarantee target intensity. For the RT, patients used resistance bands of different levels. The program also included balance exercises and fall prevention strategies during the warm-up phase.
- Time: each session lasted 60 min, including warm-up, AT, RT, and cool-down. The training protocol duration was 12 weeks.
- Volume: the prescribed workload consisted of three weekly sessions (total 36 sessions), including two RT circuits repeated 2–3 times, with 10–12 repetitions per exercise, and approximately 15 min of aerobic training.
- Progression: exercise progression was indicated to be individualized according to baseline functional status, perceived exertion, and clinical tolerance, with planned adjustment over the 12-week intervention every four weeks. Progression started with a gradual increase in training volume (repetitions, sets, circuit repetitions, and exercise duration), followed by increases in band resistance for RT and intensity for AT. Exercise intensity was maintained within the target range (RPE 3–4/10) and adapted according to individual tolerance.

*Phase 3—APE Protocol Administration*

The kinesiologist implemented the exercise program according to the FITT-VP prescription provided by the sports medicine physician, adapting it based on individual functional evaluations for the APE + LPD group. Details of the APE protocol and physical assessments are described below.

### 2.1. APE Combined Training Protocol

All patients randomized in the APE + LPD group performed a 12-week combined training protocol three times/week, 60 min each session. The training session was supervised by the kinesiologist through Microsoft Teams video calls. Patients were divided into small subgroups, with a maximum of three patients per video call for adequate supervision and safety. To ensure technical feasibility, participants were contacted before the first session to verify access to the online platform and resolve potential connection issues. At the beginning of the intervention, they received instructions to set up a safe home-based training environment, including guidance on device positioning and camera setup. An initial environmental assessment (visual inspection of the training space) was conducted to guarantee adequate visibility, safety, and proper exercise execution. Participants were also asked to connect approximately 10 min before each session to verify device setup and connectivity. In addition, they were trained to use the Borg CR10 scale and to monitor HR, either via a personal smartwatch or by manual pulse assessment. The sessions were structured as follows:

*Warm-up Phase (15 min)*

The aim of the warm-up phase was to activate the entire body, focusing on the parts that were most stimulated during the training session. Exercises included joint mobility, balance, and proprioception. This phase was mostly performed without equipment, with sticks and towels used if necessary. Some of the exercises included classic arm circles or combined half squats and calf raises, tandem walks, heel and toe walks, and mono- and bi-podalic balance exercises.

*RT phase (20 min)*

RT was organized in circuit-fashion, involving upper and lower limbs, with specific objectives for each session. Day 1: pushing exercises; Day 2: pulling exercises; Day 3: combined push and pull exercises, with the aim to improve muscle mass and strength and enhance functional capacity and overall physical performance. RT was organized into two circuits of four exercises, progressively repeated 2–3 times over the intervention period. Repetitions were typically progressed from 8–10 to 10–12, with exercises performed in accordance with the FITT-VP prescription (RPE 3–4 on the Borg CR10 scale). For this phase, exercises were performed using body weights and a CORENGTH Training Elastic Band (Supplementary Materials S1). Resistance level and the appropriate color of the elastic band (i.e., green 15 kg; yellow 25 kg; orange 35 kg) were determined considering the patient’s initial physical condition. This was established by testing the maximum number of repetitions for the “Biceps Curl” during the functional assessments at T_0_. For example, if the patient performed the biceps curl test with a medium-resistance band (yellow) and reached exhaustion after 18 repetitions, the number of repetitions for 60–80% of the maximum load would vary from 11 to 15 repetitions (minimum repetitions to plan a workout). If, after the test, the elastic band was too light to achieve minimum repetitions, a higher- resistance band was used. Conversely, if the resistance was too high and the number of repetitions too low, a lower resistance band was used. Some of the exercises included squats, static lunges, wall squats, bridges, chest press, wall push-ups, bicep curls, and crunches. Detailed progression criteria are provided in Supplementary Materials S2.

*AT Phase (15 min)*

This phase was performed with music at 120–123 bpm. Intensity was set at 65–70% of HRR in accordance with FITT-VP prescription. Due to limited space in the home, exercises performed on the spot were preferred, such as marching on the spot, free kicks, V-steps, squat kicks, and side steps with rotation. AT was progressively adjusted over the intervention period by increasing exercise duration and intensity, as detailed in Supplementary Materials S2.

*Cool-Down (10 min)*

The last part of the training involved stretching of the main muscle groups involved during the session. Each stretching position was maintained for 30 s, accompanied by correct breathing.

*Program Adaptation and Safety Considerations*

Exercise administration was adapted considering CKD-related limitations and individual clinical conditions. RT exercises were functionally adapted to reduce mechanical and cardiovascular strain. Exercises associated with high pressor responses, such as prolonged isometric contractions, were avoided or modified. Lower-limb exercises such as squats were initially performed with external support (e.g., chair-assisted squats) and progressively advanced as patient capacity improved. Particular attention was paid to postural transitions, avoiding frequent alternation between clinostat and orthostatic positions to reduce hemodynamic stress and the risk of orthostatic intolerance. Exercises were therefore organized to ensure gradual transitions between positions, minimizing the risk of dizziness or blood pressure instability. Similarly, AT was initially introduced using interval training methods to facilitate patient adaptation to continuous exercise. Over time, the activity was progressively transitioned into continuous aerobic exercise lasting up to 15 min, according to individual tolerance. Specific precautions were adopted to minimize excessive cardiovascular load, particularly in patients with hypertension. Overhead movements associated with excessive increase in HR were introduced gradually and generally postponed to the later stages of the program.

All adaptations were performed according to patients’ tolerance, perceived exertion, and clinical condition, ensuring both safety and progressive overload. Specifically, the Borg CR10 scale and HR were recorded at the end of each RT circuit, during the AT phase (midpoint and endpoint), and at the end of the entire session. These measures were used to guide real-time adjustments and to inform progression of the training program over time.

### 2.2. Assessments

The study protocol lasted a total of 12 weeks. All enrolled patients were evaluated at baseline (T_0_) before the beginning of the study and after 12 weeks (T_1_). Each patient repeated the same assessments conducted at T_0_. No changes in drug therapy were made to the enrolled patients during the study.

### 2.3. Clinical Assessments

Laboratory parameters were monitored at each time point of the study (T_0_ and T_1_). These included blood glucose, azotemia, creatinine, electrolytes (sodium, potassium, phosphorus, calcium), triglycerides, total cholesterol, low-density lipoprotein (LDL)—cholesterol, high-density lipoprotein (HDL)—cholesterol, venous bicarbonate, 24-h urine analysis (azoturia and sodiuria), and inflammatory markers (erythrocyte sedimentation rate- ESR and *C*-reactive protein- CRP and those derived from the complete blood count).

Dietary protein intake was estimated from 24-h urine nitrogen excretion levels using the Maroni and Mitch equation [29,30], while the patients’ daily intake of both sodium and salt was estimated from 24-h urine sodium excretion levels [31].

All parameters were analyzed using Dimension Vista 1500 (Siemens Healthineers AG, Forchheim, Germany) and Roche Modular P800 (Roche Diagnostics, Monza–MB, Italy) for the lipid profile. ESR and CRP were assessed via IMMULITE 2000 XPi Immunoassay System (Siemens Healthineers AG, Forchheim, Germany).

### 2.4. Body Composition Assessment and Ultrasonographic Examination

Patients’ anthropometric parameters, including body weight (kg), height (m), and body mass index (BMI, calculated as weight/height^2^), were detected at T_0_ and T_1_.

Bioelectric impedance analysis (BIA) was performed using the EGF Plus^®^ (Estor, Pero, MI, Italy), based on bioelectrical impedance vector analysis (BIVA) technology, at 50 kHz frequency. The related software Bodygram PLUS Enterprise was used to assess the patients’ body composition, and the following parameters were recorded: phase angle (PhA) in degree, total body water (TBW) in %, fat-free mass (FFM) in %, fat mass (FM) in %, body cell mass (BCM) in %, skeletal mass index (SMI), and basal metabolism in kcal. The BIA was performed according to standardized procedures [32].

QRFT was assessed in all patients using ultrasonographic examination. The measurements were performed with B-mode ultrasonography employing a 7.5 MHz transducer and an Esaote MyLab70 XVision (Genova, Italy) system equipped with an LA523 linear probe (Esaote, Genova, Italy). To minimize intra-operator variability, all assessments were conducted by the same experienced healthcare professional. The procedure consists of placing the probe perpendicular to the longitudinal axis of the muscle, with a generous layer of gel and minimal external pressure to avoid muscle compression. Three bilateral measurements were taken in the supine position, with both knees extended, at two standardized points: the midpoint between the anterosuperior iliac spine and the superior pole of the patella and the junction of the inferior third and two superior thirds of the quadriceps muscle along the same axis [33,34,35]. To standardize the measurement at the time of body composition assessment, all patients fasted for at least 8 h from food and beverages (excluding plain water). They were instructed to ensure adequate hydration in the preceding 24 h but to avoid drinking large quantities of water in close proximity to the examination. Their bladders were emptied. Moreover, they were instructed not to engage in physical activity in the preceding 24 h. The analysis was carried out outside the menstrual cycle for women of childbearing age.

### 2.5. Strength and Physical Performance Assessment

At enrolment (T_0_) and after 12 weeks (T_1_), patients underwent functional tests to evaluate muscle strength and physical performance. Before data collection, each participant was allowed to practice each test once to become familiar with the task, following standardized instructions provided by the assessor. Standardized verbal encouragement was used for all participants. To minimize fatigue and its potential influence on test performance, a rest interval of 3 min was provided between tests, and tests were administered in a fixed order. All baseline and follow-up assessments were performed by the same kinesiologist.

HGST muscle strength was measured with a Jamar Plus dynamometer (Ferreromed, Venaria Reale–TO, Italy), performing three repetitions per arm and recording maximum grip strength for both limbs. Cut-off values were <27 kg for men and <16 kg for women [36].

SPPB physical performance was assessed through three tests: gait speed (4 m walking), lower limb strength and function (5 times chair sit-to-stand), and balance (joined feet, semi-tandem, and tandem stance for 10 s) [37].

The stair climb power test (SCPT) assessed lower body strength, power, and functional performance. Patients were instructed to climb 10 steps as quickly as possible without running or jumping. Timing began when the patient touched the first step and ended when both feet reached the last step. Each patient completed two trials, with a 2-min recovery period between them [38].

The six-minute walk test (6MWT) was used to evaluate functional capacity. Patients walked as fast as possible in 6 min on a 30-m flat surface, with their heart rate monitored every minute. At the end of the test, fatigue was assessed using the Borg CR-10 Scale [39].

The sit and reach test (SRT) was used to assess hamstring and lumbar flexibility. The patient sat on the floor with their legs extended forward with flat knees. The kinesiologist placed a box against their feet, while the patient bent forward without flexing their legs. The position was held for 2 s, and distance was measured by the kinesiologist. The test was repeated three times, and the best result recorded [40].

The Back Scratch test (BScT) was performed to assess the active range of shoulder motion. The upper limb that is brought up performs a combined movement of flexion, extra-rotation, and abduction, while the other that is brought down performs a combined movement of extension, extra-rotation, and adduction. The distance between the two behind the back was recorded in centimeters [41].

### 2.6. Questionnaires

SF-36 questionnaire was used to assess the patients’ HRQoL and was administered at T_0_ and T_1_ time-points through face-to-face interviews with the patients. The SF-36 questionnaire evaluates the patients’ HRQoL through nine domains: physical functioning, physical health-related role limitations, emotional role limitations, energy/fatigue, emotional well-being, social functioning, pain, general health, and health change. Each domain generates a score from 0 to 100, expressed as a percentage, where higher scores indicate better health status [42].

The PREDIMED questionnaire was used to exclude potential biases arising from variations in the quality of foods included in the prescribed LPD, which was designed to adhere as closely as possible to the Mediterranean dietary pattern. Therefore, the PREDIMED questionnaire allowed us to assign to each patient a score at T_0_ and T_1_, based on the frequency of consumption of key Mediterranean diet foods. The PREDIMED questionnaire is based on a total score ranging from 0 to 14. Patients were classified into three groups: minimal adherence (≤5 points), medium adherence (6–9 points), and maximum adherence (≥10 points) [43].

Food diaries and 24-h recall were used as a part of remote counseling sessions, carried out every six weeks, to record all foods and beverages consumed at the time of intake over a specified period, including detailed information on portion sizes, preparation methods, and food composition [44].

### 2.7. Compliance Assessment

Compliance was analyzed through the number of attendances by each participant at supervised lessons provided for under the protocol. Compliance was expressed as a percentage of overall lessons. Possible dropout of participants and timing of this dropout were also recorded, as well as adverse events related to the protocol.

### 2.8. Statistical Analysis

All the collected data were entered into an Excel spreadsheet (Microsoft, Redmond, WA, USA). Statistical analysis was performed using a linear mixed-effects model to assess the effects of group, time, and their interaction (group × time). Normality of residuals was evaluated using the Shapiro–Wilk test.

Secondary exploratory analyses were conducted only in the presence of a significant group × time interaction. Within-group comparisons were performed using a paired Student’s *t*-test or Wilcoxon signed-rank test, as appropriate.

Intention-to-treat analysis was applied to maintain the integrity of randomization and to ensure that the results reflected the effectiveness of the intervention.

A *p*-value < 0.05 was considered statistically significant. Results with non-significant *p*-values are shown in the Supplementary Materials.

This article reports the preliminary data analysis performed on approximately 66.7% of the entire sample involved in the study. Further analyses will be performed at the end of the study.

All statistical analyses were performed using GraphPad Prism version 11.0 (GraphPad Software, San Diego, CA, USA).

## 3. Results

A total of 40 CKD patients were enrolled and divided into two homogeneous groups for age and BMI (Table 2), as follows: an APE + LPD group and an LPD group. Compliance with the exercise intervention was high, with all participants of the APE + LPD group attending at least 85% of the sessions scheduled over the 12-week period (30 of 36 sessions). Technical issues were minimal and primarily limited to initial sessions. A preliminary familiarization phase, including device setup and connection testing, was conducted, and participants were scheduled to connect with the kinesiologist before each session to verify proper connectivity and resolve potential issues in a timely manner. As a result, no sessions were missed due to technical failures and continuity of the intervention was maintained. No major adverse events were reported during the intervention.

The two groups of patients were assessed at baseline (T_0_) and 12 weeks after the start of the study (T_1_).

**Figure 3:**
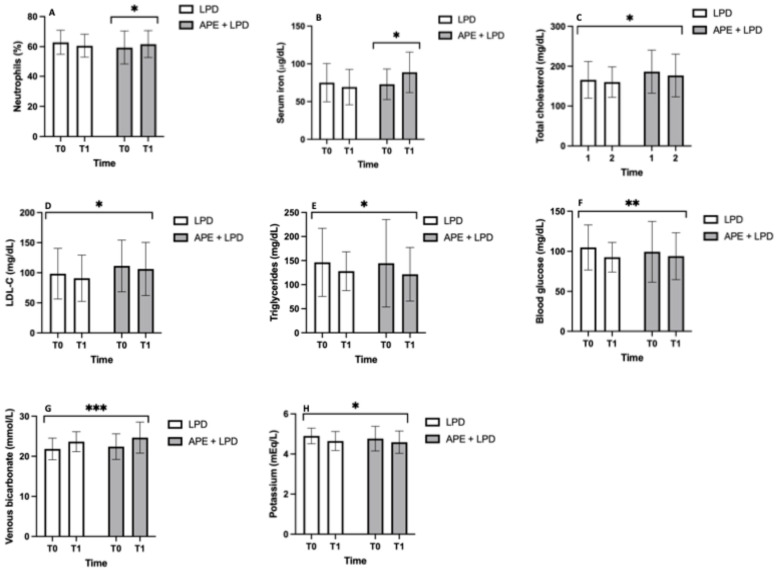
Laboratory parameters. (**A**) Neutrophils, % and their statistically significant changes only in the APE + LPD group. (**B**) Serum iron, μg/dL and its statistically significant changes only in the APE + LPD group. (**C**) Total cholesterol, mg/dL and its statistically significant changes in both groups of treatment. (**D**) LDL-c, mg/dL and its statistically significant changes in both groups of treatment. (**E**) Triglycerides, mg/dL and its statistically significant changes in both groups of treatment. (**F**) Blood glucose, mg/dL and its statistically significant changes in both groups of treatment. (**G**) Venous bicarbonate, mmol/L and its statistically significant changes in both groups of treatment. (**H**) Potassium mEq/L and its statistically significant changes in both groups of treatment. The data are reported as mean ± standard deviation. Abbreviations: APE, adapted physical exercise; LDL-c, low-density lipoprotein cholesterol; LPD, low-protein diet. *, *p* < 0.05; **, *p* < 0.01; ***, *p* < 0.001.

Complete laboratory parameters, including iron profile and pro-inflammatory biomarkers, are reported in Supplementary Materials S3–S6. Figure 3 illustrates the laboratory parameters of the two study groups. Neutrophils (%) and serum iron levels showed a significant group/time interaction (*p* < 0.05). Post hoc analysis demonstrated a significant improvement only in the APE + LPD group, whereas no significant changes were observed in the LPD group (panel A: LPD, 62.8 ± 7.7 to 60.3 ± 7.8%; APE + LPD, 59.4 ± 10.2 to 61.6 ± 8.9. Panel B: LPD, 75.1 ± 25.4 to 69.3 ± 23.4; APE + LPD, 72.9 ± 20.3 to 88.7 ± 26.8 μg/dL).

In contrast, total cholesterol, low-density lipoprotein cholesterol (LDL-c), triglycerides, blood glucose, and potassium significantly decreased over time in both groups (main effect of time, *p* ranging from <0.05 to <0.01) while venous bicarbonate significantly increased (main effect of time, *p* < 0.001), with no significant interaction between group and time (panel C: LPD, 169.2 ± 45.9 to 157.4 ± 48.2 mg/dL; APE + LPD, 186.1 ± 51.9 to 176.6 ± 50.6 mg/dL. Panel D: LPD, 100.8 ± 42.2 to 86.9 ± 39.0 mg/dL; APE + LPD, 111.4 ± 43.7 to 106.3 ± 41.5 mg/dL. Panel E: LPD, 145.5 ± 70.5 to 130.6 ± 39.8 mg/dL; APE + LPD, 144.5 ± 80.4 to 121.6 ± 52.3 mg/dL. Panel F: LPD, 104.9 ± 29.1 to 94.2 ± 18.9 mg/dL; APE + LPD, 99.5 ± 36.2 to 94.0 ± 27.3 mg/dL. Panel G: LPD, 21.8 ± 2.7 to 23.6 ± 2.5 mmol/L; APE + LPD, 22.4 ± 3.2 to 24.6 ± 3.9 mmol/L. Panel H: LPD, 4.9 ± 0.4 to 4.6 ± 0.5 mEq/L; APE + LPD, 4.8 ± 0.7 to 4.6 ± 0.6 mEq/L).

**Figure 4:**
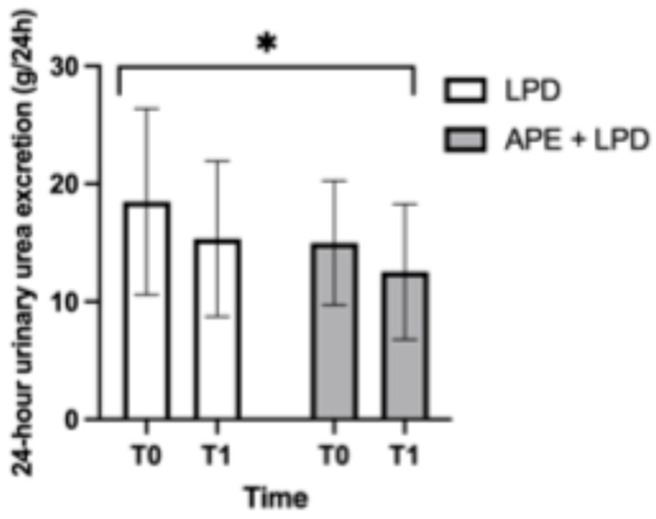
24-h urinary urea excretion. The data are reported as mean ± standard deviation. Abbreviations: APE, adapted physical exercise; LPD, low-protein diet. *, *p* < 0.05.

Figure 4 shows 24-h urinary urea excretion of the two study groups. This measure of 24-h urinary urea excretion decreased over time in both groups (main effect of time, *p* < 0.05), with no significant interactions between group and time (LPD, 18.5 ± 7.9 to 14.9 ± 5.9 g/kg/day; APE + LPD, 15.0 ± 6.3 to 12.5 ± 5.5 g/kg/day).

**Figure 5:**
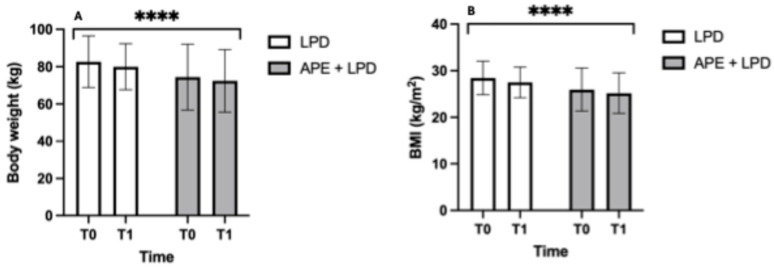
Anthropometric parameters. (**A**) Body weight, kg and its statistically significant changes in both groups of treatment. (**B**) BMI, kg/m^2^ and its statistically significant changes in both groups of treatment. The data are reported as mean ± standard deviation. Abbreviations: APE, adapted physical exercise; BMI, body mass index; LPD, low-protein diet. ****, *p* < 0.0001.

Figure 5 illustrates the anthropometric parameters of the two study groups. Both groups showed a significant reduction in body weight over the 12 weeks of the study (main effect of time, *p* < 0.0001) and in BMI, while no significant differences were observed between the groups (panel A: LPD, 82.6 ± 13.8 to 79.9 ± 12.3 kg; APE + LPD, 74.4 ± 17.7 to 72.4 ± 16.8 kg. Panel B: LPD, 28.5 ± 3.6 to 27.5 ± 3.3 kg/m^2^; APE + LPD, 26.0 ± 4.6 to 25.2 ± 4.3 kg/m^2^).

**Figure 6:**
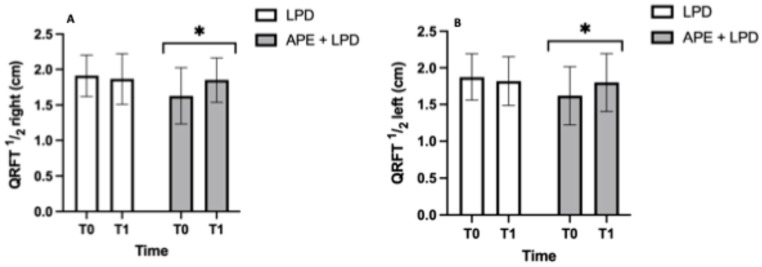
Body composition parameters. (**A**) QRFT ½ right, cm and its statistically significant changes in APE+ LPD group. (**B**) QRFT ½ left, cm and its statistically significant changes in APE+ LPD group. The data are reported as mean ± standard deviation. Abbreviations: APE, adapted physical exercise; LPD, low-protein diet; QRFT, quadriceps rectus femoris thickness; Rz, reactance. *, *p* < 0.05.

Figure 6 shows the body composition parameters of the two study groups. Ultrasound evaluation of QRFT ½ reference point of both the right and left thighs revealed a significant group/time interaction (*p* < 0.05), with post hoc analyses confirming a significant increase only in the APE + LPD group (Panel B: LPD, 1.9 ± 0.3 to 1.9 ± 0.4 cm; APE + LPD, 1.6 ± 0.4 to 1.8 ± 0.3 cm. Panel C: LPD, 1.9 ± 0.3 to 1.8 ± 0.3 cm; APE + LPD, 1.6 ± 0.4 to 1.8 ± 0.4 cm).

**Figure 7:**
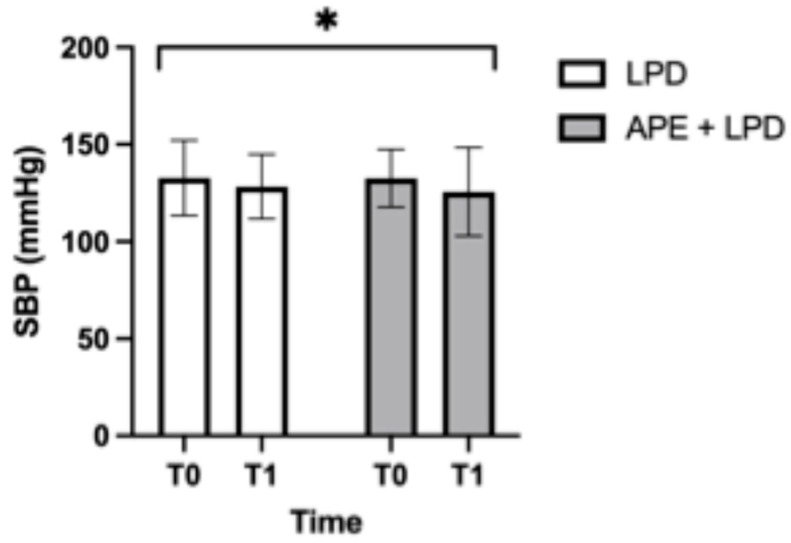
Systolic blood pressure values. The data are reported as mean ± standard deviation. Abbreviations: APE, adapted physical exercise; LPD, low-protein diet; SBP, systolic blood pressure. *, *p* < 0.05.

Figure 7 illustrates the systolic blood pressure (SBP) values of the two study groups. SBP decreased significantly over time in both groups (main effect of time, *p* < 0.05), with no significant interaction between group and time (LPD, 133 ± 19 to 128 ± 16 mmHg; APE + LPD, 132 ± 15 to 126 ± 23 mmHg).

**Figure 8:**
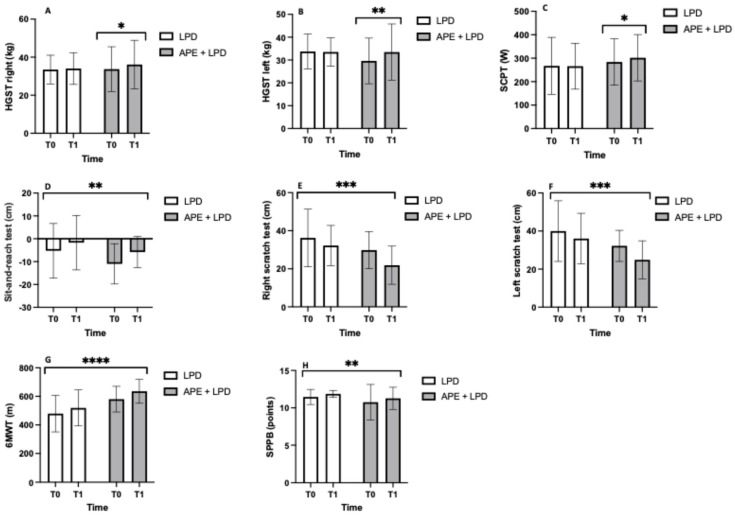
Muscle strength and physical performance parameters. (**A**) HGST right, kg and its statistically significant changes only in the APE + LPD group. (**B**) HGST left, kg and its statistically significant changes only in the APE + LPD group. (**C**) SCPT (W) and its and its statistically significant changes only in the APE + LPD group. (**D**) Sit-and-reach test, cm and its statistically significant changes in both groups of treatment. (**E**) Right scratch test, cm and its statistically significant changes in both groups of treatment. (**F**) Left scratch test, cm and its statistically significant changes in both groups of treatment. (**G**) 6MWT, m and its statistically significant changes in both groups of treatment. (**H**) SPPB, points its statistically significant changes in both groups of treatment. The data are reported as mean ± standard deviation. Abbreviations: 6MWT, six-minute walking test; APE, adapted physical exercise; HGST, handgrip strength test; LPD, low-protein diet, SCPT, stair climb power test, SPPB short physical performance battery. *, *p* < 0.05; **, *p* < 0.01; ***, *p* < 0.001; ****, *p* < 0.0001.

Figure 8 shows the parameters relating to muscle strength and physical performance for the two study groups. Assessment of muscle strength highlights physical performance as a significant interaction between group and time for the HGST for both arms and for SCPT (for both tests, *p* ranging from <0.05 to <0.01), with post hoc analyses confirming the obtained results only in the APE + LPD group (Panel A: LPD 33.5 ± 7.6 to 34.0 ± 8.3 kg; APE + LPD, 33.7 ± 11.7 to 36.1 ± 12.7 kg. Panel B: LPD, 33.7 ± 7.6 to 33.5 ± 6.2 kg; APE + LPD 30.7 ± 10.6 to 33.7 ± 12.6 kg. Panel C: LPD, 267.4 ± 121.4 to 265.9 ± 97.7 W; APE + LPD 284.0 ± 99.3 to 301.9 ± 99.0 W). Conversely, physical performance showed a significant change over time in both groups (main effect of time, *p* ranging from <0.01 to 0.0001) in the SRT, the right and left ScT, the 6 MWT and SPPB (Panel D: LPD −5.23 ± 11.9 to −1.7 ± 11.9 cm; APE + LPD, −11.0 ± 8.8 to −5.8 ± 6.8 cm. Panel E: LPD, 36.2 ± 15.1 to 32.2 ± 10.6 cm; APE + LPD, 29.8 ± 9.8 to 21.9 to 10.0 cm. Panel F: LPD, 40.0 ± 15.9 to 36.0 ± 13.3 cm; APE + LPD 32.2 ± 8.2 to 24.9 ± 10.0 cm. Panel G: LPD, 479.0 ± 128.0 to 519.1 ± 126.3 m; APE + LPD, 580.3 ± 89.9 to 635.6 ± 83.9 m. Panel H: LPD, 11 ± 1 to 12 ± 0.5 points; APE + LPD, 10 ± 2 to 11 ± 1 points).

**Figure 9:**
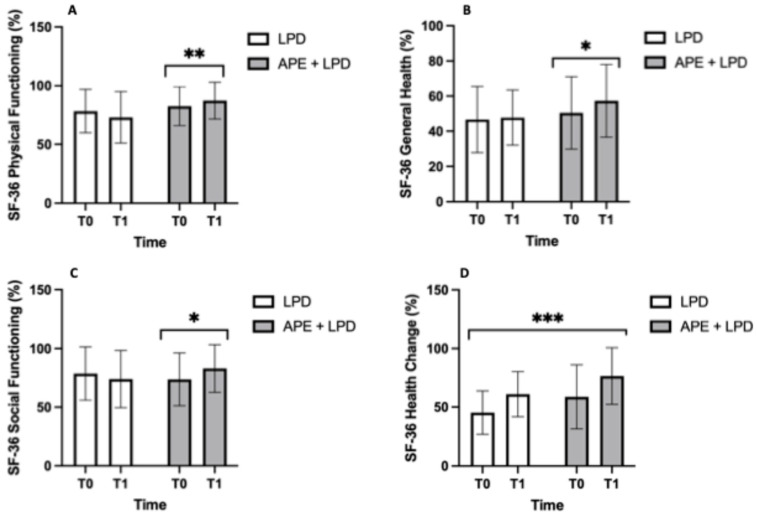
Domains of the Short Form Health Survey 36. (**A**) SF-36 Physical Functioning, % and its statistically significant changes only in the APE + LPD group. (**B**) SF-36 General Health, % and its statistically significant changes only in the APE + LPD group. (**C**) SF-36 Social Functioning, % and its statistically significant changes only in the APE + LPD group. (**D**) SF-36 Health Change, % and its statistically significant changes only in the APE + LPD group. The data are reported as mean ± standard deviation. Abbreviations: APE, adapted physical exercise; LPD, low-protein diet; SF, Short Form Health Survey. *, *p* < 0.05; **, *p* < 0.01; ***, *p* < 0.001.

Figure 9 illustrates the domains of SF-36 for the two study groups. The SF-36 questionnaire revealed a significant interaction between group and time for physical functioning, general health, and social functioning domains (*p* ranging from <0.05 to <0.01), with post hoc analyses confirming a significant improvement in HRQoL only in the APE + LPD group. In contrast, the SF-36 health change domain revealed a significant change over time in both groups (main effect of time, *p* ranging from <0.001).

**Figure 10:**
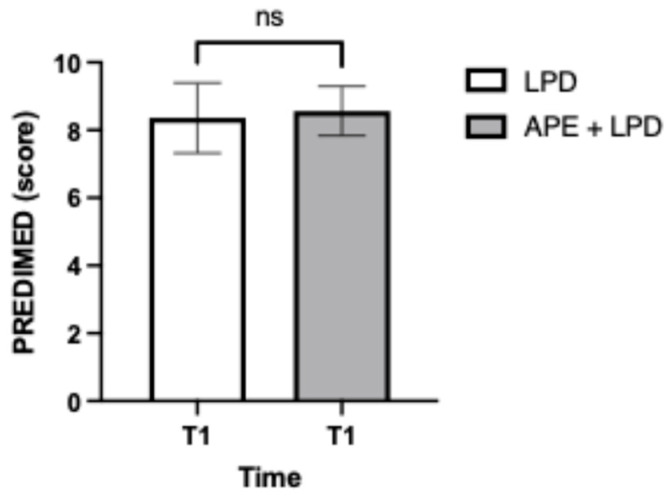
PREvención con Dieta MEDiterránea questionnaire. The data are reported as mean ± standard deviation. Abbreviations: APE, adapted physical exercise; LPD, low-protein diet; ns, not significant; PREDIMED, PREvención con Dieta MEDiterránea.

Figure 10 shows the scores obtained by the two study groups in the PREDIMED questionnaire. At the end of the study (T_1_), no statistically significant difference was found between the two study groups in the PREDIMED questionnaire scores (LPD, 8.4 ± 1; APE + LPD 8.6 ± 0.7).

Other parameters that did not show statistically significant differences between APE + LPD and LPD are shown in Supplementary Materials S3–S6.

## 4. Discussion

The preliminary results obtained in this randomized clinical trial conducted on conservative CKD patients highlighted that the combination of APE and LPD is more effective in muscle-related outcomes and HRQoL and, moreover, seems to suggest a positive modulation of the immune system beyond the metabolic effects observed with LPD alone.

While both groups exhibited significant ameliorations in anthropometric and lipid and glucose metabolism parameters, only the APE + LPD group demonstrated significant enhancements for QRFT ½ reference point, HGST, specific SF-36 questionnaire domains (i.e., physical functioning, general health, and social functioning), neutrophil percentage, and serum iron levels.

These findings suggest that the tailored LPD intervention likely represents the primary driver of metabolic improvements, whereas, when it is combined with a structured APE program, it provides additional benefits by targeting muscle-related outcomes and HRQoL in CKD patients. In this regard, the KDIGO 2024 guidelines emphasize that controlled protein restriction, when appropriately monitored, may reduce metabolic burden while preserving nutritional status in CKD patients [21]. Consistently, a reduction in body weight, BMI, and nPCR observed in both groups confirms adherence to the prescribed LPD. Moreover, although body weight decreased significantly during the intervention, it remained within the baseline BMI range, suggesting that the dietary intervention was appropriately calibrated and nutritionally adequate.

In addition, both groups exhibited improvements in lipid profile, glycemic control, SBP, and venous bicarbonate values. The rise in bicarbonate concentration is particularly relevant in CKD patients, where metabolic acidosis has been demonstrated to contribute to muscle catabolism and disease progression [45,46]. Previous analyses from the Modification of Diet in Renal Disease (MDRD) study and subsequent trials have demonstrated that dietary modulation of acid load can positively influence serum bicarbonate levels and metabolic parameters [47,48]. Similarly, electrolyte levels, including serum potassium, were maintained during the study.

Alongside metabolic effects, LPD seems to support overall physical capacity, as evidenced by improvements in the 6MWT and SPPB scores observed in both groups. These functional gains may partly reflect the benefits of DNT, such as enhanced acid–base balance and metabolic control, and modest reductions in body weight, in line with previous study in CKD patients following well-monitored LPD [14,49,50].

Therefore, the observed improvements likely reflect the overall benefits of DNT, which include personalized LPD, dietary counselling, and structured multidisciplinary follow-up. In contrast, the most clinically relevant gains in muscle hypertrophy and strength were observed only in patients who participated in the structured APE program, highlighting that APE provides unique and additive benefits that complement the beneficial effects of dietary management.

Specifically, ultrasound assessment revealed a significant increase in QRFT ½ reference point, exclusively in the APE + LPD group. In fact, skeletal muscle wasting and sarcopenia are highly prevalent in CKD and are associated with adverse outcomes, including all-cause mortality and functional decline. Recent studies have validated ultrasound measurement of QRFT as a reliable and prognostically meaningful marker in CKD patient populations [51,52].

The improvements observed in the APE + LPD group were achieved through a combined exercise protocol, incorporating both aerobic and resistance training, with particular emphasis on muscle strengthening. This structured approach appears to counteract the catabolic effects of CKD and mitigate potential risks associated with protein restriction, aligning with evidence that resistance-focused interventions enhance muscle hypertrophy and functional capacity in CKD patients [53,54].

At the same time, HGST significantly improved only in the APE + LPD group. HGST is widely recognized as a surrogate for global muscle strength and nutritional status. Its lower values have been independently associated with increased mortality and faster progression of CKD versus ESKD [55,56,57]. Meta-analyses also confirm that reduced HGST predicts mortality in patients under renal replacement therapy [56,57]. The selective improvement observed in the APE + LPD group therefore represents not only a functional gain but also a new tool for detecting better outcomes in CKD patients.

The HRQoL findings further support the added value of APE. Significant improvements were observed in the SF-36 domains of physical functioning, general health, and social functioning. HRQoL is frequently impaired in CKD, and it is strongly associated with morbidity and hospitalization risk. Previous randomized trials have demonstrated that structured exercise programs improve SF-36 physical domains in CKD patients under conservative treatment [53,54]. The parallel improvement in muscle strength and thickness observed in our study suggests that enhanced physical capacity may mediate perceived health gains.

An additional interesting finding concerns the significant improvement for neutrophil (expressed in percentage) and serum iron levels, which ws limited to the APE + LPD group. CKD is characterized by chronic low-grade inflammation and dysregulated iron metabolism, often mediated by elevated hepcidin levels and functional iron deficiency. Physical exercise has been proposed to influence inflammatory pathways and iron homeostasis, although biochemical mechanisms remain incompletely understood [58,59]. Previous studies have suggested that moderate-intensity physical activity may affect iron metabolism and oxidative stress markers at the molecular level [56]. Similarly, exercise has been shown to induce transient changes in neutrophil dynamics, reflecting acute innate immune activation followed by adaptive responses with repeated exposure [57]. In the present study, the observed changes in neutrophil percentage and serum iron may reflect a potential physiological adaptation to the combined intervention. These findings appear promising and are consistent with previous evidence; however, they require further confirmation in studies that include a more comprehensive panel of inflammatory biomarkers and a longer follow-up period. Overall, taken together, these findings support the hypothesis that combining LPD with a structured APE can induce additive benefits on muscle strength and structure and HRQoL.

This study has limitations, including the relatively small sample size and short follow-up duration. However, its strengths include randomized prospective design, homogeneous baseline characteristics, muscle ultrasound assessments, and multidimensional evaluation including metabolic, functional, and HRQoL outcomes.

Future larger-scale and longer-duration trials should investigate dose–response relationships of APE, including inflammatory and iron regulatory biomarkers and evaluating long-term renal and clinical endpoints.

## 5. Conclusions

Tailored nutritional intervention with a well-monitored LPD provides essential metabolic control and supports physical functioning maintenance in CKD patients under conservative therapy. However, the addition of a structured APE program, combining aerobic and resistance training, seems to offer targeted benefits in muscle strength and structure, and in HRQoL. This integrated approach could not only complement the metabolic effects of dietary therapy and contribute to functional preservation, reducing the risk of CKD-related complications but also enhancing patients’ perception of their health, positively impacting self-efficacy and overall HRQoL.

**Figure 11:**
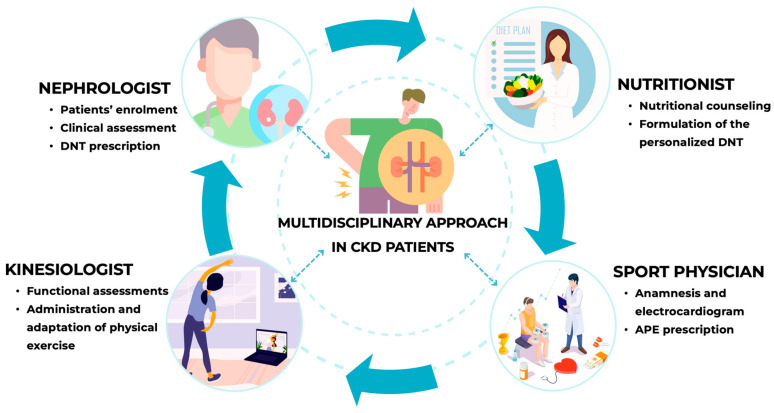
A virtuous multidisciplinary approach to chronic kidney disease patient. Abbreviations: APE, adapted physical exercise; CKD, chronic kidney disease; DNT, dietary–nutritional therapy.

These preliminary data highlight the important role of a multidisciplinary approach in CKD which should integrate the expertise of kinesiologists and sports physicians alongside nephrologists and nutritionists (Figure 11). This framework could be a starting point for new therapeutical strategies based on patients’ features. In fact, this approach would allow even more accurately tailoring of the most appropriate integrative therapeutical strategy for each patient.

## Figures and Tables

**Table 1:** Characteristics of the study population divided between the two groups.

	APE + LPD Group			LPD Group		
	G3b	G4	G5	G3b	G4	G5
**Stage at baseline (number of patients)**	8	10	2	8	10	2
**Mean e-GFR at baseline for each stage (mL/min/1.73 m^2^)**	34.4 ± 6.7 *	27.7 ± 6.2 *	13.5 ± 2.1 *	36.7 ± 5.3 *	25.1 ± 7.4 *	13.1 ± 2.1 *
**Key comorbidities (%)**	Diabetes mellitus (45%), arterial hypertension (80%), dyslipidemia (79%), secondary anemia (62%), secondary hyperparathyroidism (81%)			Diabetes mellitus (47%), arterial hypertension (82%), dyslipidemia (84%), anemia (60%), secondary hyperparathyroidism (76%)		
**Medication profile (%)**	Antihypertensive drugs (80%), SGLT2-i (85%), insulin (20%), other oral hypoglycemic drugs (10%), hypouricemic drugs (70%), statins (85%), other hypolipemic drugs (20%), iron supplementation (76%), vitamin D3 analogues (74%), erythropoietin (20%)			Antihypertensive drugs (78%), SGLT2-i (86%), insulin (24%), other oral hypoglycemic drugs (6%), hypouricemic drugs (64%), statins (89%), other hypolipemic drugs (16%), iron supplementation (78%), vitamin D3 analogues (77%), erythropoietin (18%)		

* Data are expressed as mean ± standard deviation. Abbreviation SGLT2-i: sodium/glucose cotransporter 2-inhibitors.

**Table 2:** Main epidemiological and anthropometric features of the study populations.

	APE + LPD Group	LPD Group	*p*-Value
N°	20	20	-
Sex (M/F)	16/4	12/8	-
Age (years)	62 ± 10 ^a^	63 ± 9 ^a^	0.8603
BMI (kg/m^2^)	26.4 ± 4.7 ^a^	28.5 ± 3.6 ^a^	0.0903

**^a^** Data expressed as mean ± standard deviation. *p*-values were calculated using the Mann–Whitney U test. Abbreviations: APE, adapted physical exercise; BMI, body mass index; F, female; LPD, low-protein diet; M, male.

## Data Availability

The datasets presented in this article are not immediately available as they contain patients’ personal data. Requests for access to the datasets should be addressed to the corresponding author.

## Supplementary Materials

The following supporting information can be downloaded at: https://www.mdpi.com/article/10.3390/nu18101557/s1, Supplementary Materials S1—Types of elastic bands subdivided by colour according to the resistance offered; Supplementary Materials S2—APE combined training protocol progression criteria; Supplementary Materials S3—Routine laboratory parameters; Supplementary Materials S4—Biomarkers and indices of inflammation; Supplementary Materials S5—Anthropometric and body composition parameters; Supplementary Materials S6—Blood pressure parameters and hearth rate.
