# Supplementary material for: Is Adapted Physical Exercise an Innovative Adjuvant Approach to Combine with Low-Protein Diet in Chronic Kidney Disease?
Source: Nutrients. 2026 May 14;18(10):1557. doi: 10.3390/nu18101557 (PMC13209451; doi:10.3390/nu18101557)
Supplement: Supplementary file 1 [file nutrients-18-01557-s001.zip › nutrients-4241545-supplementary.pdf]

SUPPLEMENTARY MATERIALS

- **Materials S1** - Types of elastic bands subdivided by colour according to the resistance offered.
- **Materials S2** - APA combined training protocol progression criteria.
- **Materials S3** – Routine laboratory parameters.
- **Materials S4 –** Biomarkers and indices of inflammation.
- **Materials S5 –** Anthropometric and body composition parameters.
- **Materials S6 –** Blood pressure parameters and hearth rate.

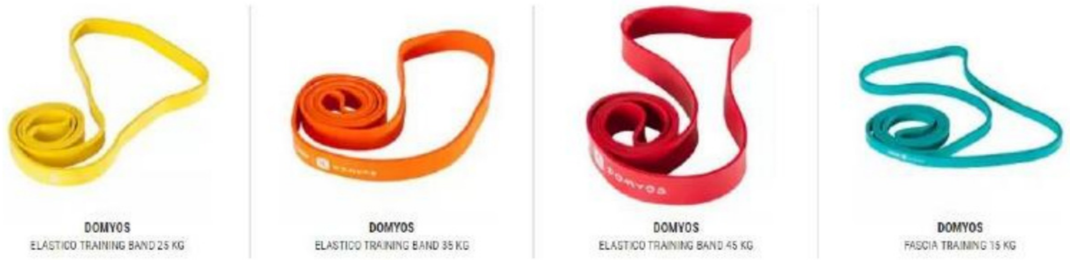

Supplementary Materials S1—Types of elastic bands subdivided by colour according to the resistance offered.

Weeks	Resistance Training	Circuit Structure	Aerobic Training	Progression Focus
**Weeks 1–2**	Familiarization phase; focus on technique and movement control. 1–2 sets × 8–10 reps (RPE 3-4 Borg CR10). Low resistance bands.	1–2 circuits × 2 reps	Intermittent bouts to facilitate adaptation to continuous exercise. Total duration: ~10 min. Intensity: ~65% HRR.	Neuromuscular adaptation and safe exercise learning.
**Weeks 3–4**	Maintain technique; improve execution and control. 10 reps.	2 circuits × 2 reps	Transition toward continuous aerobic exercise. Duration: 5-10 min. Intensity maintained (~65% HRR).	Increase aerobic volume; consolidate RT technique.
**Weeks 5–6**	Increase repetitions to 10–12. RPE maintained.	2 circuits × 2 reps	Continuous aerobic exercise. Duration: 10-15 min.	Increase muscular endurance and training volume.

Weeks	Resistance Training	Circuit Structure	Aerobic Training	Progression Focus
**Weeks 7–8**	Maintain repetitions; focus on time under tension. (4-Borg CR10)	2 circuits × 2–3 reps	Increase intensity to ~70% HRR. Duration: 15 min.	Increase cardiovascular stimulus and overall workload.
**Weeks 9–10**	Increase resistance (higher elastic band) or reduction of band length. Reduce repetitions to 8–10.	2 circuits × 3 reps	Maintain intensity (~70% HRR).	Shift toward strength stimulus.
**Weeks 11–12**	Increase repetitions to 10–12 with higher resistance.	2 circuits × 3 reps	Maintain duration and intensity (~70% HRR).	Consolidation of strength and total workload.
**Note:** When progression in resistance band was not feasible, the duration of each progression phase was extended, allowing patients to remain longer at a given workload level before advancing, according to individual tolerance and clinical condition.				

Supplementary Materials S2—APE combined training protocol progression criteria. Abbreviations: APE, adapted physical exercise; HRR, Heart Rate Reserve; min, minutes; reps, repetitions, RPE, rate of perceived exertion.

	APE+LPD group		LPD group		P value for group/time interaction
**Parameters**	**T_0**	**T_1**	**T_0**	**T_1**	
**Hb (g/dL)**	13.3 ± 1.6	13.3 ± 1.7	13.4 ± 1.8	13.2 ± 1.9	0.2380
**Lymphocytes (%)**	28.4 ± 8.9	26.8 ± 8.3	26.3 ± 7.2	27.6 ± 6.1	0.1158
**Monocytes (%)**	7.3 ± 2.4	6.8 ± 1.7	7.0 ± 2.2	7.7 ± 1.9	0.0866
**Eosinophils (%)**	2.7 ± 1.8	3.2 ± 2.0	3.7 ± 1.4	3.8 ± 1.8	0.3041
**Basophils (%)**	0.8 ± 0.3	0.9 ± 0.4	0.6 ± 0.4	0.6 ± 0.3	0.7527
**Creatinine (mg/dL)**	2.3 ± 0.9	2.4 ± 1.3	2.7 ± 1.2	2.7 ± 1.6	0.4349
**e-GFR* (mL/min/1.73 m²)**	30.4 ± 10.3	30.7 ± 11.5	27.8 ± 12.1	29.9 ± 15.0	0.1973
**Azotemia (mg/dL)**	69.4 ± 32.2	67.1 ± 34.0	82.7 ± 34.6	74.2 ± 42.9	0.3190
**Sodium (mEq/L)**	141.7 ± 3.0	141.7 ± 3.5	140.9 ± 2.6	139.4 ± 2.6	0.1742
**Calcium (mEq/L)**	9.6 ± 0.4	9.6 ± 0.3	9.4 ± 0.6	9.1 ± 0.5	0.3317
**Phosphorus (mEq/L)**	3.8 ± 0.9	3.6 ± 0.5	3.6 ± 0.7	3.4 ± 0.7	0.9674
**HDL-cholesterol (mg/dL)**	50.8 ± 15.8	49.1 ± 18.5	46.2 ± 11.3	47.1 ± 14.2	0.3187
**Uricemia**	5.9 ± 2.0	5.9 ± 2.2	6.00 ± 1.3	5.8 ± 1.4	0.6297
**Ferritin (ng/mL)**	107.2 ± 101.5	121.6 ± 91.2	107.2 ± 78.4	114.4 ± 88.6	0.7317
**Transferrin (mg/dL)**	241.4 ± 39.8	248.7 ± 39.6	244.0 ± 41.7	233.1 ±72.0	0.3446

**Sodiuria (mmol/24h)**	118.9 ± 57.6	105.8 ± 63.5	150.0 ± 57.7	142.5 ± 71.2	0.9433
**ACR (mg/g creatinine)**	412.3 ± 366.7	647.2 ± 336.9	289.0 ± 227.4	126.8 ± 109.0	0.7027

Supplementary Materials S3—Routine laboratory parameters. *eGFR calculated according to CKD-EPI formula. The data are reported as mean ± standard deviation. Abbreviations: ACR, albumin-creatinine ratio; APE, adapted physical exercise; e-GFR, estimated glomerular filtration rate; Hb, hemoglobin; LPD, low-protein diet.

	**APE+LPD group**		**LPD group**		**P value for group/time interaction**
**Parameters**	**T_0**	**T_1**	**T_0**	**T_1**	
**CRP (mg/dL)**	1.9 ± 3.2	2.4 ± 4.4	1.0 ± 1.2	1.2 ± 2.9	0.5690
**ESR (mm/h)**	21.7 ± 16.8	24.3 ± 18.9	22.5 ± 14.04	20.9 ± 14.3	0.2237
**Platelet/ lymphocyte**	116.0 ± 21.7	110.1± 26.7	112.0 ± 15.7	112.1± 20.7	0.1034
**Neutrophil/ lymphocyte**	1.27 ± 0.34	1.43 ± 0.41	1.25 ± 0.14	1.27 ± 0.22	0.0675
**Lymphocyte/ monocyte**	4.8 ± 1.51	5.1 ± 1.39	5.3 ± 2.11	4.9 ± 2.39	0.4327
**Lymphocyte (n/mm3)**	2.1 ± 0.3	2.4 ± 0.6	1.9 ± 0.6	2.3 ± 0.3	0.0875

Supplementary Materials S4—Biomarkers and indices of inflammation. The data are reported as mean ± standard deviation. Abbreviations: APE, adapted physical exercise; CRP, C-reactive protein; ESR, erythrocyte sedimentation rate; LPD, low-protein diet.

	**APE+LPD group**		**LPD group**		**P value for group/time interaction**
**Parameters**	**T_0**	**T_1**	**T_0**	**T_1**	
**Rz (ohm-Ω)**	462.8 ± 60.2	470.7 ± 61.5	456.8 ± 63.2	479.7 ± 61.5	0.6380
**Xc (ohm-Ω)**	43.9 ± 18.1	48.7 ± 9.5	44.8 ± 8.9	48.8 ± 9.5	0.8372
**Phase angle (°)**	5.4 ± 1.4	5.3 ± 0.6	5.6 ± 0.98	5.8 ± 1.0	0.4871
**TBW (%)**	56.2 ± 6.5	56.5 ± 8.1	55.8 ± 6.2	55.5 ± 6.5	0.7044
**ECW (%)**	48.9 ± 5.9	48.7 ± 3.4	49.0 ± 5.9	46.9 ± 4.4	0.6089
**ICW (%)**	51.1 ± 5.9	51.3 ± 3.4	52.4 ± 5.9	53.1 ± 4.4	0.7906
**FM (%)**	24.3 ± 9.0	23.7 ± 8.4	25.3 ± 9.0	24.9 ± 8.4	0.9714
**FFM (%)**	74.1 ± 12.9	76.3 ± 10.9	74.8 ± 9.2	75.0 ± 8.4	0.4060
**BCM (%)**	48.4 ± 9.3	50.5 ± 3.6	51.3 ± 6.3	52.5 ± 4.8	0.6975
**BMR (Kcal/day)**	1570 ± 239	1556 ± 212	1673 ± 223	1663 ± 183	0.7703

**QRFT ²/₃ left (cm)**	1.5 ± 0.3	1.5 ± 0.4	1.5 ± 0.3	1.5 ± 0.3	0.6879
**QRFT ²/₃ right (cm)**	1.5 ± 0.4	1.6 ± 0.3	1.5 ± 0.3	1.5 ± 0.3	0.3532

Supplementary Materials S5—Anthropometric and body composition parameters;. The data are reported as mean ± standard deviation. Abbreviations: APE, adapted physical exercise; BCM, body cell mass; BMR, basal metabolic rate; ECW, extra-cellular water; FFM, free fat mass; FM, fat mass; ICW, intra-cellular water; LPD, low-protein diet; QRF ²/₃ left, quadriceps rectus femoris thickness two-thirds left; QRFT ²/₃ right, quadriceps rectus femoris two-thirds right; Rz, Resistance; TBW, Total Body Water; Xc, Reactance.

	**APE+LPD group**		**LPD group**		**P value for group/time interaction**
**Parameter**	**T_0**	**T_1**	**T_0**	**T_1**	
DBP	85 ± 13	82 ± 15	81 ± 12	78 ± 10	0.6978

Supplementary Materials S6—Blood pressure parameters and hearth rate. The data are reported as mean ± standard deviation. Abbreviations: APE, adapted physical exercise; LPD, low-protein diet; DBP, diastolic blood pressure.
